# Impact of Obesity and Hyperglycemia on Placental Mitochondria

**DOI:** 10.1155/2018/2378189

**Published:** 2018-08-14

**Authors:** Chiara Mandò, Gaia Maria Anelli, Chiara Novielli, Paola Panina-Bordignon, Maddalena Massari, Martina Ilaria Mazzocco, Irene Cetin

**Affiliations:** ^1^Department of Biomedical and Clinical Sciences, Unit of Obstetrics and Gynecology, ASST Fatebenefratelli Sacco University Hospital, Università degli Studi di Milano, Via G. B. Grassi 74, 20157 Milano, Italy; ^2^Division of Genetics and Cell Biology, IRCCS Ospedale San Raffaele, Via Olgettina 60, 20132 Milano, Italy

## Abstract

A lipotoxic placental environment is recognized in maternal obesity, with increased inflammation and oxidative stress. These changes might alter mitochondrial function, with excessive production of reactive oxygen species, in a vicious cycle leading to placental dysfunction and impaired pregnancy outcomes. Here, we hypothesize that maternal pregestational body mass index (BMI) and glycemic levels can alter placental mitochondria. We measured mitochondrial DNA (mtDNA, real-time PCR) and morphology (electron microscopy) in placentas of forty-seven singleton pregnancies at elective cesarean section. Thirty-seven women were normoglycemic: twenty-one normal-weight women, NW, and sixteen obese women, OB/GDM(−). Ten obese women had gestational diabetes mellitus, OB/GDM(+). OB/GDM(−) presented higher mtDNA levels versus NW, suggesting increased mitochondrial biogenesis in the normoglycemic obese group. These mitochondria showed similar morphology to NW. On the contrary, in OB/GDM(+), mtDNA was not significantly increased versus NW. Nevertheless, mitochondria showed morphological abnormalities, indicating impaired functionality. The metabolic response of the placenta to impairment in obese pregnancies can possibly vary depending on several parameters, resulting in opposite strains acting when insulin resistance of GDM occurs in the obese environment, characterized by inflammation and oxidative stress. Therefore, mitochondrial alterations represent a feature of obese pregnancies with changes in placental energetics that possibly can affect pregnancy outcomes.

## 1. Introduction

The placenta is a metabolically active organ with multiple functions connecting the mother and the fetus for a successful outcome of pregnancy [1].

Mitochondrial oxidative phosphorylation and substrate oxidation represent the main energy source for placental function [2]. Therefore, mitochondrial function or dysfunction plays an important role in metabolic health and cellular fate [3]. In the human and rodent placenta, both nutritional and hypoxic stresses can alter mitochondrial function [4–10], with changes in mitochondrial biogenesis, function, and morphology leading to placental dysfunction. Placental alterations can affect fetal metabolism and development possibly leading to higher risk of developing disease in the future adult [11].

In the last decade, obesity has become a global problem [12]. Maternal obesity (MO) is expanding exponentially worldwide to almost epidemic proportions, with an additional 5–10% of pregnant women with diabetes, representing a significant risk factor for adverse pregnancy outcomes [13–17] with both immediate and long-term consequences [11, 18–22]. However, molecular mechanisms underlying programming effects have been only partially identified. Impaired placental transfer and metabolism of energy substrates in maternal obesity and/or diabetes mellitus have been reported [23, 24]. A lipotoxic placental environment is indeed recognized in maternal obesity, with an altered metabolome profile, increased inflammation and oxidative stress, and decreased regulators of angiogenesis [25–28]. This might alter mitochondrial function, resulting in excessive production of reactive oxygen species and oxidative stress, in a vicious cycle leading to placental dysfunction and impaired pregnancy outcomes.

In this study, we addressed the hypothesis that maternal pregestational body mass index (BMI) and glycemic levels can alter placental mitochondria, by measuring mitochondrial content and morphology in term placentas sampled at elective cesarean section.

## 2. Materials and Methods

Pregnant women were enrolled in the Unit of Obstetrics and Gynecology of the Luigi Sacco Hospital in Milan.

The study protocol was approved by the local Institutional Review Board (Luigi Sacco Hospital Ethical Committee), and all participants gave their informed consent.

### 2.1. Population

Only Caucasian women with singleton spontaneous pregnancy and delivering at term by elective cesarean section were included in this study. Cesarean sections before labor were performed for breech presentation, repeated caesarean section, or maternal request. Exclusion criteria were represented by maternal-fetal infections or autoimmune diseases, maternal smoking and drug-alcohol abuse, fetal malformations, chromosomal disorders, preeclampsia, and intrauterine growth restriction.

Forty-seven pregnant women were eligible for the study.

Thirty-seven presented normal glycemia values based on an oral glucose tolerance test (OGTT—75 g) [29]. Among them, twenty-one were within normal weight (NW; 18.5 ≤ BMI < 25 kg/m^2^) and sixteen were obese (OB/GDM(−); BMI ≥ 30 kg/m^2^) according to their pregestational BMI [30].

Ten women were diagnosed with gestational diabetes mellitus (GDM) according to the OGTT at 24–28 weeks of gestation, and all of them were obese (OB/GDM(+)). Women with GDM underwent daily checks of glycemia.

All women were given nutritional and lifestyle advice and recommendations on weight gain during pregnancy following IOM guidelines, depending on pregestational BMI [30].

Obese patients had regular specific checkups in a dedicated antenatal clinic, where they received specific dietary indication to support the control of their gestational weight gain and their glycemia levels. No patient needed insulin therapy.

NW women had physiological pregnancies with a normal intrauterine growth and appropriate for gestational age birth weight according to reference ranges for the Italian population [31].

### 2.2. Data Collection

Maternal medical history, demographic, anthropometric, obstetric, and neonatal data were recorded at recruitment and after cesarean delivery.

Maternal hemoglobin was measured at 34–36 weeks. Maternal fasting glycemia was obtained from the first value of the OGTT performed between 24 and 28 weeks.

### 2.3. Sample Collection and Processing

Human placentas were collected immediately after elective cesarean section, in the absence of labor. Placentas were weighed after discarding membranes and cord from the disc, and biometric measurements were performed as previously described [18].

After removing the maternal decidua, placental biopsies (~1 cm^3^) were sized from different cotyledons [32] midway between the cord insertion and placental border. Placental villi were then washed in PBS (Dulbecco's phosphate-buffered solution; Euroclone, Milano, Italy) and immediately frozen in liquid nitrogen to be stored at −80°C until mtDNA analysis or alternatively were fixed with 2.5% glutaraldehyde for electron microscopy.

#### 2.3.1. Placenta mtDNA Content

Frozen placental fragments (90 mg) were minced in a TRIzol reagent (Roche Diagnostics, Indianapolis, IN, USA) with a Potter homogenizer. Total DNA was isolated from this mixture with a chemical procedure, following the manufacturer's instructions. DNA concentrations were measured by a NanoDrop ND1000 spectrophotometer (NanoDrop Technologies; Wilmington, DE, USA).

Mitochondrial DNA (mtDNA) content was assessed by real-time PCR, normalizing levels of a mitochondrial gene (cytochrome-*β*, CYB) to those of a single-copy nuclear gene (RNase-P) (2^−ΔCq^ method). Briefly, 30 nanograms of total DNA was analyzed in triplicate with TaqMan assays (CYB: Hs02596867_s1 and RNase-P: 4316844) by 7500 Fast Real-Time PCR (Applied Biosystems, Thermo Fisher Scientific; Carlsbad, CA, USA); Cq values with standard deviation exceeding 0.25 were excluded.

#### 2.3.2. Electron Microscopy

Cells were fixed with 2.5% glutaraldehyde in 100 mM cacodylate buffer pH 7.4 for 1 hour at room temperature. After several washes in cacodylate buffer, cells were postfixed with 1% osmium tetroxide and 1.5% potassium ferrocyanide in 100 mM cacodylate buffer pH 7.4 for 1 hour on ice. After a rinse in dH_2_O, samples were en bloc stained in 0.5% uranyl acetate overnight and dehydrated in increasing concentrations of ethanol and finally embedded in Epon. Samples were cured at 60°C in an oven for 48 h. Epon blocks were sectioned using a Leica EM UC7 ultramicrotome (Leica Microsystems, UK). Ultrathin sections (70 nm) were contrasted with 2% uranyl acetate and Sato's lead solutions and observed with a LEO 912AB Zeiss Transmission Electron Microscope (Carl Zeiss, Oberkochen, Germany). Digital micrographs were taken with a 2k × 2k bottom-mounted slow-scan ProScan camera (ProScan, Lagerlechfeld, Germany) controlled by the EsivisionPro 3.2 software (Soft Imaging System, Münster, Germany).

### 2.4. Statistical Analyses

Data are presented as mean ± standard error.

Maternal, fetal, and molecular data were compared among study groups by one-way analysis of variance (ANOVA), having preliminarily verified that no serious statistical violations occurred. Tukey HSD test was then run as a post hoc test.

A two-way between-group ANOVA was conducted to explore the impact of maternal BMI/GDM and fetal sex (independent variables), as individual or joint effect, on placental levels of mtDNA (dependent variable).

Chi-square analyses were performed to compare anemia frequencies among groups, using Yates continuity correction.

Correlations describing the strength and direction of the relationships between 2 parameters were assessed using the Pearson product-moment correlation.

All statistical tests were 2-sided, and *p* values < 0.05 were considered statistically significant. Statistical analysis was performed using SPSS (version 24.00, IBM Statistics; Armonk, NY, USA).

## 3. Results

### 3.1. Characteristics of the Study Population

Maternal, fetal and placental data are reported in Table 1.

According to inclusion criteria, pregestational BMI was significantly different among groups (*F*(2, 46) = 78.52, *p* ≤ 0.001), being higher in the two obese groups compared to NW. Obese women gained on average less weight during pregnancy compared to normal weight, mostly remaining within IOM recommended limits for gestational weight gain during pregnancy of obese women [30]. As expected, maternal fasting glycemia was significantly different among groups (*F*(2, 46) = 3.71, *p* = 0.03), with OB/GDM(+) showing higher levels compared to normoglycemic groups (Tukey HSD test, *p* = 0.03). Hemoglobin levels were lower in the two OB groups compared to NW, though not significant. However, anemia (Hb < 11.0 g/dl) frequency was higher in OB, resulting more than two-fold higher in OB/GDM(−) (56%) and in OB/GDM(+) (50%) than in NW (25%) subjects.

Maternal age, gestational age, and fetal weight did not differ among groups.

There was a statistically significant difference in placental weight (*F*(2, 46) = 4.75, *p* = 0.01). Post hoc comparisons using the Tukey HSD test indicated that the mean score for OB/GDM(+) was significantly higher compared to the NW group (*p* = 0.01).

Placental efficiency (fetal/placental weight ratio) was significantly different among groups (*F*(2, 46) = 3.29, *p* = 0.04), with OB/GDM(+) showing decreased placental efficiency compared to NW (*p* = 0.03).

**Figure 1:**
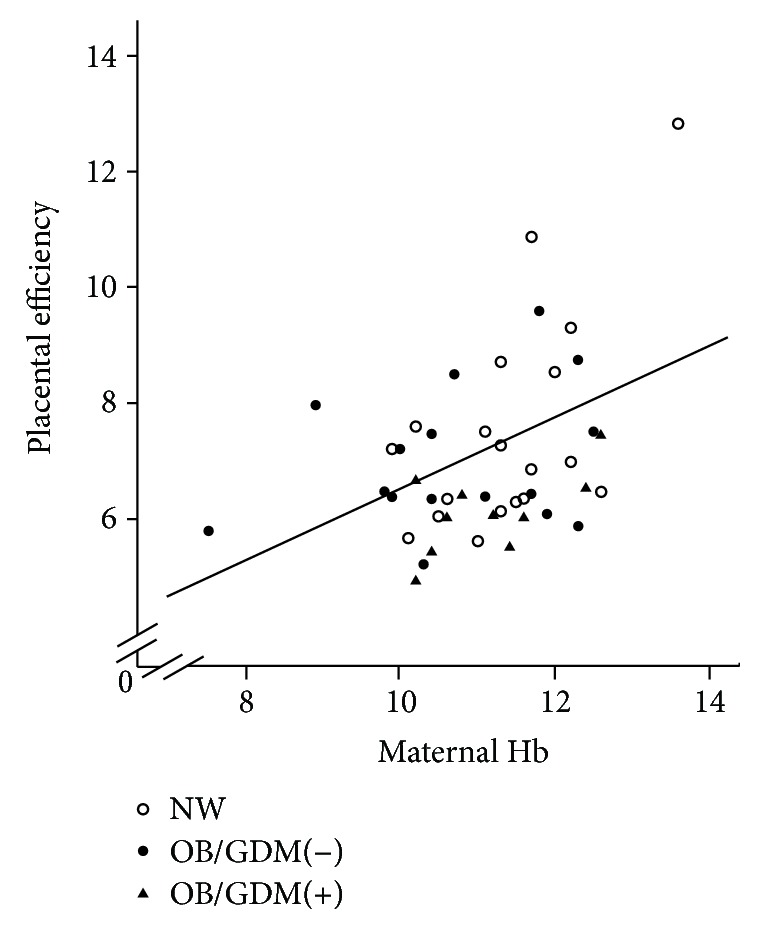
Significant correlation between placental efficiency and maternal hemoglobin (*p* = 0.005, *r* = 0.412). NW: normal-weight women; OB/GDM(−): obese women without a diagnosis of GDM; OB/GDM(+): obese women with GDM.

In our study population, placental efficiency was significantly and positively correlated with maternal Hb (*p* = 0.005, *r* = 0.412, Figure 1) and with gestational age (*p* = 0.001, *r* = 0.457), while it was negatively correlated with placental thickness (*p* = 0.03, *r* = −0.336). Maternal Hb also positively correlated with gestational age (*p* = 0.004, *r* = 0.422) and negatively with placental weight (*p* = 0.03, *r* = −0.323) (data not shown).

Among the analyzed pregnancies, 23 carried male fetuses (15 of NW, 6 of OB/GDM(−), and 2 of OB/GDM(+) mothers) and 24 carried female fetuses (6 of NW, 10 of OB/GDM(−), and 8 of OB/GDM(+) mothers).

### 3.2. mtDNA Content in Placental Tissue

**Figure 2:**
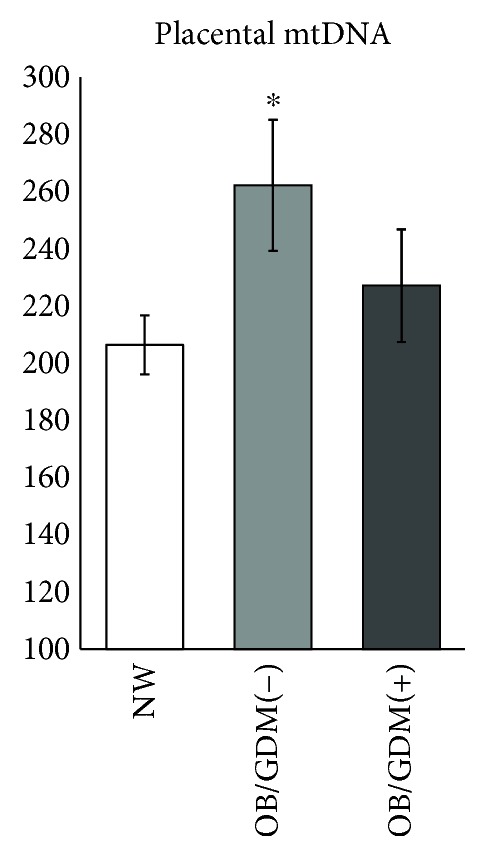
Placental mtDNA levels. ^∗^*p* = 0.047 versus NW, Tukey HSD test. NW: normal-weight women; OB/GDM(−): obese women without a diagnosis of GDM; OB/GDM(+): obese women with GDM.

A one-way between-group analysis of variance was conducted to explore the impact of obesity and GDM on levels of mitochondrial DNA. There was a statistically significant difference among groups in mtDNA levels (*F*(2, 46) = 3.03, *p* = 0.49). Post hoc comparisons using the Tukey HSD test indicated that the mean score for the OB/GDM(−) group was significantly higher compared to NW (*p* = 0.047), while OB/GDM(+) was not (Figure 2).

**Figure 3:**
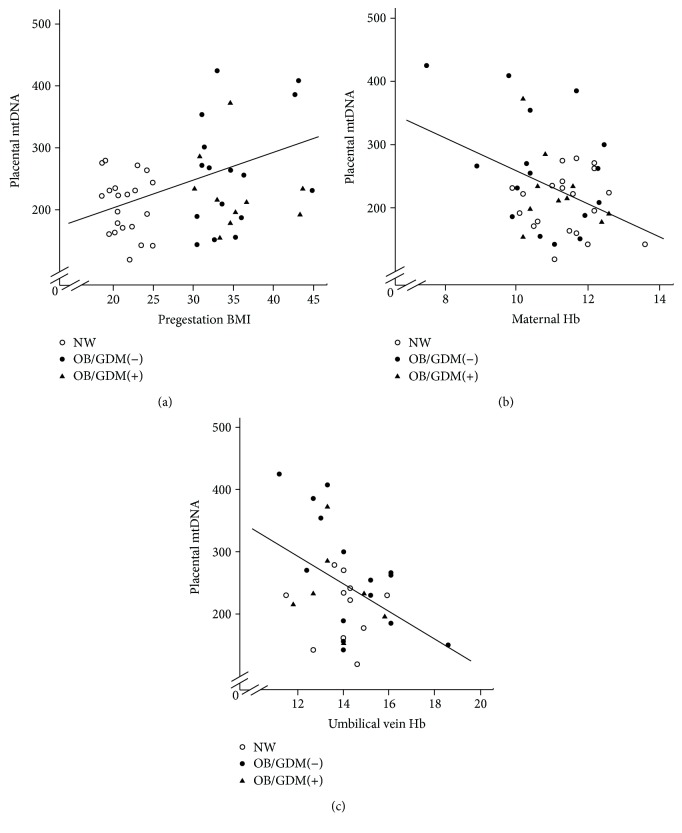
(a) Correlation between placental mtDNA and maternal pregestational BMI. The correlation is significant in patients without a diagnosis of GDM, indicated by the regression line (*p* = 0.010, *r* = 0.419). (b). Correlation between placental mtDNA and maternal hemoglobin (*p* = 0.011, *r* = −0.373). (c). Correlation between placental mtDNA and umbilical vein hemoglobin (*p* = 0.019, *r* = −0.406). NW: normal-weight women; OB/GDM(−): obese women without a diagnosis of GDM; OB/GDM(+): obese women with GDM.

Figure 3(a) shows the relation between mtDNA levels and pregestational BMI in the study population. There was a significant correlation in patients without a diagnosis of GDM, indicated by the regression line (*p* = 0.010, *r* = 0.419), while no significant correlation was found in OB patients with GDM. mtDNA also negatively correlated with maternal Hb (*p* = 0.011, *r* = −0.373) (Figure 3(b)) and umbilical vein Hb (*p* = 0.019, *r* = −0.406) (Figure 3(c)).

There were no differences in mtDNA levels depending on fetal sex. The two-way between-group analysis of variance showed that the interaction effect between fetal sex and maternal pregestational BMI was not statistically significant (*F*(2, 46) = 0.61, *p* = 0.94) (data not shown).

### 3.3. Electron Microscopic Analysis of Syncytiotrophoblast

**Figure 4:**
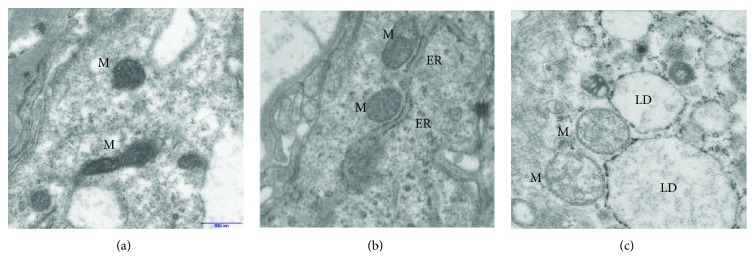
Electron microscopy of term placenta villi showing representative sections of syncytiotrophobast cells from NW (a), OB/GDM(−) (b), and OB/GDM(+) (c) term placentas. M: mitochondria; ER: endoplasmic reticulum; LD: lipid droplet. NW: normal-weight women; OB/GDM(−): obese women without a diagnosis of GDM; OB/GDM(+): obese women with GDM.

The mitochondrial profiles in the syncytiotrophoblast of both NW and OB/GDM(−) placentas were round or elongated with a very dense matrix and similar structure of the cristae (Figures 4(a) and 4(b)). In contrast, syncytiotrophoblast mitochondria in OB/GDM(+) placentas displayed morphological abnormalities, showing a matrix with very low density and vesicle-like or disrupted cristae, forming an irregular pattern (Figure 4(c)).

## 4. Discussion

Recently, maternal obesity has been associated with a lipotoxic placental environment, with increased placental lipids, inflammation, and oxidative stress, together with a less efficient fetal/placental ratio and altered metabolome profile [18, 23, 25, 27]. The cellular stress characterizing this maternal environment may adversely affect placental development and function possibly altering fetal growth and development. Indeed, oxidative stress is one of the hallmark responses to intracellular lipid overload. High levels of free fatty acids impact the mitochondrial (mt) membrane structure, causing the release of reactive oxygen species (ROS) that can react with macromolecules and damage intracellular membranes and DNA [33]. These alterations can in turn affect mitochondrial structure and function, in a vicious cycle of mitochondrial abnormalities and ROS formation, possibly representing a key mechanism of placental dysfunction in a disease condition.

Several animal models of MO report mt dysfunctions in pancreatic islets, liver, or skeletal muscle of the offspring [34, 35]. However, maternal obesity and diabetes are not always associated with obvious fetal distress, and the possible placental adaptation may explain it [24].

In this study, we addressed the hypothesis that placental mitochondria in pregnancy can be altered by elevated maternal BMI and/or by metabolic alterations occurring in gestational diabetes mellitus.

We studied placentas at term only delivered by elective cesarean section, in order to avoid possible alterations of mitochondrial content or function due to labor [36]. Obese patients were followed during pregnancy with a specific counseling including nutritional and lifestyle advices. This resulted in lower gestational weight gain compared to normal weight patients, as recommended by IOM guidelines [30]. Our study population was also carefully selected by excluding women carrying further conditions possibly affecting mitochondrial characteristics, such as maternal smoking or drug-alcohol abuse and maternal or fetal pathologies. Obese women with GDM were included, in order to evaluate the additional effect of increased glycemia to placental mitochondrial features.

### 4.1. mtDNA in Obese Pregnancies without GDM

mtDNA levels are largely recognized as a measure of the mitochondrial content [4, 37, 38]. We found higher levels of mtDNA, accounting for the increased mitochondrial content in placental cells of women with an obese pregestational BMI without a diagnosis of GDM (Figure 2). The morphology of mitochondria in the syncytiotrophoblast of the OB/GDM(−) group was overall similar to the NW group, suggesting no alterations in mitochondrial function (Figures 4(a) and 4(b)). A compensatory increase in mitochondrial biogenesis can be explained by the endocrine stimuli due to high intracellular fatty acid levels and oxidative stress occurring in the lipotoxic environment of obese placentas [9, 34]. Indeed, altered levels of mtDNA as well as the impairment of nutrient transport systems have been reported in previous studies in the placental tissue of different pregnancy pathologies characterized by elevated oxidative stress and inflammation levels, such as intrauterine growth restriction and preeclampsia [4, 5, 39–43]. The positive correlation between placental mtDNA and maternal BMI that was observed in this study supports this hypothesis (Figure 3(a)).

Differently from our study, decreased mtDNA copy number has been previously reported in placentas of obese compared to not-obese women [9, 44]. However, different gene assays and different population criteria were employed in these studies. One of the strengths of the present study is the careful selection of a very well characterized population of uncomplicated pregnancies. We excluded any maternal or fetal infection or autoimmune disease, maternal smoking or drug-alcohol abuse, fetal malformations, chromosomal disorders, preeclampsia, and intrauterine growth restriction, all of which can affect mitochondrial biogenesis and functionality. Moreover, only Caucasian women were selected, as different mitochondrial DNA haplogroups have been identified and have been suggested to possibly contribute to the genetic component of complex disorders [45–47]. Finally, all women included in this study were counseled with nutritional and lifestyle advice and recommendations on weight gain during pregnancy, and obese patients had regular specific checkups in a dedicated antenatal clinic with specific dietary indication.

Interestingly, in a recent review on mitochondrial features in gestational disorders, Holland and colleagues reported that mitochondrial content has been found to be increased or decreased in the same pregnancy pathology by different studies [48]. These apparent differences within the same pathologies can be explained by the different severity or timing of the insult and the resulting capacity of the tissue to respond. Indeed, although in several diseases, mitochondrial biogenesis is thought to occur as a compensatory mechanism to the cell distress [4, 49–52], on the other hand, the increase of mitochondrial ROS production could damage the mitochondrial DNA and membranes, thus inhibiting the adaptive mitochondrial increase.

Therefore, the different inclusion/exclusion criteria of the studied population, together with different clinical protocols in nutritional and lifestyle advice, can lead to different results regarding the mitochondrial responses. This is also suggested in the recent study reporting increased placental mitochondrial content in early-onset but not late-onset preeclampsia [41].

### 4.2. mtDNA in Obese Pregnancies with GDM

When analyzing placentas of obese women with GDM, we did not find a significant increase in mtDNA levels (Figure 2). However, placentas of OB/GDM(+) women showed dysfunctional syncytiotrophoblast mitochondria, with morphological abnormalities. In particular, electron microscopy revealed a loss of matrix density and disorganization of inner membrane cristae (Figure 4(c)). Interestingly, also animal models of diabetes showed a reduced number of mitochondria, with abnormal morphology, associated to mitochondrial dysfunction [53]. Notably, large lipid droplets that stock lipids as energy-rich storage compounds were observed. In this context, lipid droplets may support feeble mitochondrial function by supplying fatty acids for mitochondrial *β* oxidation and protect mitochondria from lipotoxicity [54].

Our results therefore suggest that placental mitochondria of obese women with GDM do not show significant alterations in biogenesis but present altered morphology, indicating an impairment of their function.

The OB/GDM(+) group also presented decreased placental efficiency compared to NW. Indeed, GDM has been associated with impaired placental development showing villous immaturity or alterations in villous branching, as well as impaired placental angiogenesis, villous vasculature, and uteroplacental perfusion [55–57]. Oxidative stress markers have also been reported in GDM placentas [58], possibly affecting the physiology of the placental vasculature and mitochondrial morphology.

Insulin resistance and altered metabolic profiles characterize the diabetic condition. Insulin resistance has been correlated in several tissues with a decrease in mitochondrial function and mitochondrial DNA copy number, a reduction in mitochondrial fusion and increase in their fission and with alterations of mitochondrial size and density. The possible role of epigenetic regulation is emerging for these alterations [59–64]. Indeed, some authors recently hypothesized that insulin resistance acts on the expression of proteins involved in the methylation machinery of both nuclear and mitochondrial DNA, affecting the expression of genes involved in mtDNA replication, thus leading to decreased mitochondrial biogenesis [62, 65, 66]. However, other studies report either no impairment or a compensatory increase of mitochondrial function and oxidative capacity in conditions of insulin resistance [67, 68]. Hence, the relationship between mitochondria and insulin action is highly complex and there is still much to learn in this area [3].

Similarly to our results, decreased levels of placental mtDNA have been recently reported in diabetic pregnancies [69], together with lower mitochondrial respiratory chain enzyme activities [9]. In our population, different levels of placental mtDNA in obese with or without GDM may be the result of opposite strains. Maternal diabetes has been associated with a decrease of placental mitochondrial levels [9, 69], while the obese environment associated with inflammation and oxidative stress tends to promote a compensatory mitochondrial biogenesis. In the recent study, we reported comparable results in the maternal blood of obese women with or without GDM [52]. Levels of mtDNA in maternal blood may indeed result by the release of placental cell debris in the maternal circulation [70].

In addition, complex changes in the metabolic profile have been shown in obese pregnant women with or without GDM [25, 71]. White et al. recently showed that in addition to the dysregulation of glucose metabolism, GDM obese women compared with non-GDM obese women exhibited exaggerated dyslipidemic profiles prior to the GDM diagnosis, at week 17, when placentation still occurs. This possibly reflects enhanced insulin resistance in peripheral tissues of GDM women and a consequent reduced suppression of lipolysis, affecting lipid metabolism pathways. Increased insulin resistance and higher levels of lipids and lipoproteins have also been shown at mid and late pregnancy in women with GDM compared to normal glucose tolerant patients [72].

Moreover, GDM-obese women showed metabolic patterns consistent with perturbed energy pathways [25, 71]. Particularly, obese women with GDM showed increased levels of acetoacetate (likely secondary to unregulated fatty acid oxidation in mitochondria) and citrate (an early intermediate of the tricarboxylic acid cycle, occurring in mitochondria). These evidences suggest a specific metabolic milieu of GDM compared to non-GDM obese pregnant women, which can differently affect the mitochondrial function in placenta.

### 4.3. Relation between mtDNA and Maternal/Fetal Hemoglobin

Noteworthy, in our study population, we found a significant negative correlation between placental mtDNA and hemoglobin levels in the maternal and fetal blood (Figures 3(b) and 3(c)). Maternal Hb also significantly correlated with placental efficiency.

Maternal systemic hemoglobin may account for maternal nutritional status. Obese women in this study did not present significantly lower levels of maternal Hb, possibly due to the specific and regular nutritional counseling given to them during pregnancy. However, in our population anemia frequency (Hb < 11.0 g/dl) was higher in both OB subgroups compared to NW. Indeed, obesity induces a chronic, low-grade inflammation with overexpression of C-reactive protein and hepcidin [26, 52] that is negatively correlated with both maternal and cord blood iron status [73]. On the other hand, low maternal Hb may be an index of altered vascular oxygenation that can induce mitochondrial biogenesis, according to the negative correlation we found in our study population.

### 4.4. Limitations

A sexual dimorphism has been reported in several placental responses to adverse environments [13, 18, 74]. Nevertheless, in our study population, we did not find any interaction effect between the fetal sex and maternal pregestational BMI, suggesting that the BMI influence on mtDNA levels and morphology does not depend on fetal sex. However, a limited number of cases within each subgroup might lead to these results. Therefore, further investigations are needed to explore the possible effect of fetal sex on the mitochondrial content and function in placentas of obese pregnancies with or without GDM.

Although our results on placental mitochondria of OB/GDM(+) pregnancies comply with previous findings, showing lower mitochondrial copy number compared to NW placentas [9, 69], inclusion criteria of the populations analyzed in these studies were different to ours, also including type 1 and type 2 diabetes mellitus, placentas from vaginal deliveries, and different ethnic groups, thus keeping open the need of further studies investigating the consequences of insulin action on placental mitochondria in GDM pregnancies.

Moreover, whether alterations in mtDNA content of OB placental whole tissue are due to alterations in one or more placental cell types still remains to be investigated.

## 5. Conclusions

The placenta integrates nutritional and endocrine signals and arranges its metabolic phenotype to support pregnancy. The metabolic response of the placenta to impairment depends on the nature, severity, and duration of the environmental adversity [1], and in obese pregnancies, these can possibly vary depending on several parameters like maternal glycemia or the maternal nutritional status and lifestyle. However, mitochondrial alterations are a clear feature of obese pregnancies with changes in placental energetics and consumption of oxidative substrates that possibly can affect fetal delivery of nutrients and O_2_ with short- and long-term consequences on the newborn.

Understanding the differences in placental metabolic adaptation to obesity and insulin resistance might open new perspectives for therapeutic future developments [24].

## Figures and Tables

**Table 1:** Maternal, fetal and placental characteristics in the study population.

	NW, *n* = 21	OB/GDM(−), *n* = 16	OB/GDM(+), *n* = 10
Maternal data			
Age, years	35.7 ± 1.02	32.7 ± 1.26	35.7 ± 1.39
Pregestational BMI, kg/m^2^	21.5 ± 0.45	34.9 ± 1.17^∗∗∗^	35.6 ± 1.46^∗∗∗^
Fasting glycemia, mg/dl	81.4 ± 1.72	82.0 ± 1.80	90.4 ± 4.13^∗^
Hb, g/dl	11.4 ± 0.20	10.7 ± 0.34	11.1 ± 0.27
GWG, kg	10.38 ± 0.58	7.94 ± 1.06	8.50 ± 1.57
GWG to IOM advised limits, %	64.9 ± 3.65	95.5 ± 9.18	92.8 ± 17.43
Fetal & placental data at delivery			
Gestational age, weeks	39.2 ± 0.12	39.1 ± 0.07	39.1 ± 0.05
Fetal weight, g	3329.6 ± 63.1	3339.1 ± 87.8	3394.0 ± 126.0
Placental weight, g	460.6 ± 19.2	488.4 ± 22.0	559.0 ± 20.5^∗∗^
Placental efficiency	7.54 ± 0.41	7.00 ± 0.30	6.11 ± 0.23^∗^
Placental area, cm^2^	257.5 ± 15.7	243.7 ± 16.2	259.3 ± 17.7
Placental thickness, cm	1.84 ± 0.12	2.12 ± 0.15	2.25 ± 0.17
Umbilical vein Hb, g/dl	14.0 ± 0.34	14.4 ± 0.48	13.7 ± 0.51

Data are presented as mean ± standard error. Post hoc comparisons using the Tukey HSD test: ^∗^*p* ≤ 0.05, ^∗∗^*p* ≤ 0.01, ^∗∗∗^*p* ≤ 0.001 versus NW. NW: normal weight; OB/GDM(−): obese without a diagnosis of GDM; OB/GDM(+): obese with GDM; GDM: gestational diabetes mellitus; BMI: body mass index; maternal fasting glycemia: referred to the first value of the oral glucose tolerance test (OGTT); Hb: hemoglobin; GWG: gestational weight gain; IOM: Institute of Medicine; placental efficiency: fetal/placental weight ratio; placental area: (larger diameter) × (smaller diameter) × (*π*/4).

## Data Availability

Readers may access the data underlying the findings of the study by writing to the corresponding author.
